# Discussion of the Influence of Multiscale PCA Denoising Methods with Three Different Features

**DOI:** 10.3390/s22041604

**Published:** 2022-02-18

**Authors:** Chizhou Zhang, Tao Sun

**Affiliations:** 1 Electronic & Computer Science School, University of Shouthampton, Southampton SO17 1BJ, UK; cabbagebkue@163.com; 2 Electronic Information School, Wuhan University, Wuhan 430072, China

**Keywords:** EMG signal, ICA, wavelet function, signal decomposition, KNN

## Abstract

Bioinformation is information generated from biological movement. By using a variety of modern technologies, we can use this information to form a meaningful model for researchers to study. An electromyographic (EMG) signal is one type of bioinformation that is used in many areas to help people study human muscle movement. This information can help in both clinical areas and industrial areas. EMG is a very complicated signal, so processing it is vital. The processing of EMG signals is divided into collection, denoising, decomposition, feature extraction and classification steps. In this article, the wavelet denoising step and several decomposition processes are discussed to show the usage of this technique in the final classification step. At the end of the study, we find that after the wavelet denoising step, the classification accuracy, which uses the K-nearest neighbor of the independent component analysis features, improves, but the accuracy of the wavelet coefficient features and autoregression coefficient features decreases.

## 1. Introduction

An electromyographic (EMG) signal is a time-series signal that is collected mainly for clinical applications, and it has been widely used in research laboratories to meet the needs of sectors such as biomechanics and motor control [1]. After 1866, when Grace and Erlanger used an oscilloscope to show electrical signals from muscles, Cram and Steger utilized it in a clinical approach to scan a variety of muscles, and after the 1880s, when the appropriate amplifier was invented, people began to pay much more attention to EMG signals [2]. EMG signals have been investigated and widely debated over many decades. Mary described electromyographic signals as muscular activity involving muscle neurons and regulating muscle fiber inside the motor unit [3]. Based on this description and other research, the collection methods of EMG signals are divided into two types. One type is called the transcutaneous method and collects motor unit potentials (MUPs) by placing an array of electrodes on the skin. The other type, which is called the intramuscular method, requires a wire or needle electrode to be directly placed into the muscle to collect the MUPs directly [4]. The different collection methods of EMG signals mean that the processing methods are diverse. A raw EMG signal can be regarded as a time-series signal, and the features in its time and frequency domain can be obtained to perform classification. Additionally, as the number of collecting channels of the EMG signal increases, the multichannel EMG signal can be processed as a multidimensional signal process; for example, ref. [5] shows that the multichannel EMG signal is suitable for the blind source separation theory. Multidimensional signals are an important form of information. Compared with one-dimensional signals, multidimensional signals contain more energy and more information, and their accuracy can be improved through the connections between all dimensions. A common example of a two-dimensional signal is an image signal, and an example of a three-dimensional signal is a video signal [6].

A simple way to deal with a multichannel EMG signal is to deal with the average of all the signals in each channel or to use each channel’s signal to perform feature extraction; however, this loses the crosstalk between different EMG signals near each other, which causes information loss. Therefore, in this article, we illustrate some methods that are used to process multichannel EMG signals, and we compare them to normal processing methods to show the differences between the features that the different methods yield.

## 2. Materials and Methods

### 2.1. EMG Signal Components

To understand how to process EMG signals, the construction of an EMG signal needs to be discussed. The fundamental component of an EMG signal is the muscle fiber action potential (MFAP), which is the action potential caused by the excitable membrane of a muscle fiber [7]. All MFAPs in a motor unit together make up the motor unit action potential (MUAP), and many motor units fire repeatedly to maintain muscle action, which is summed to produce an EMG signal [7].

**Figure 1:**
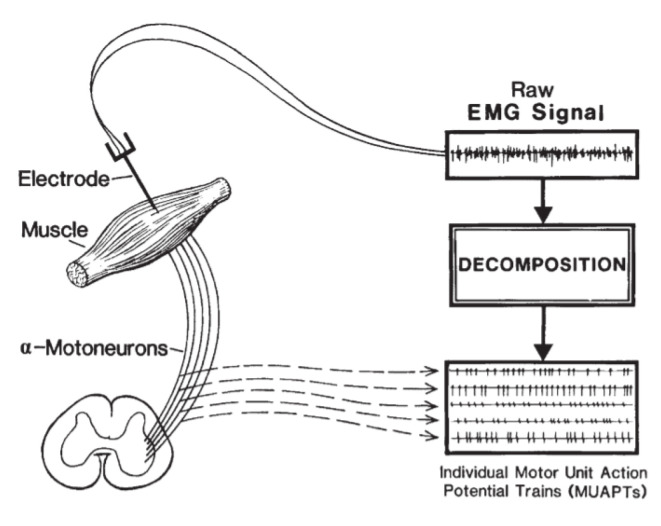
EMG signal composition and how it can be decomposed [7].

**Figure 2:**
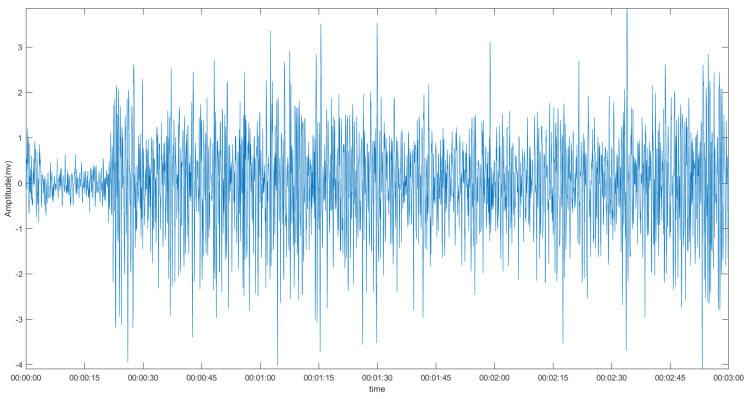
Example of a raw EMG signal.

Figure 1 shows the basic structure of an EMG signal and how it can be decomposed, and Figure 2 shows a raw EMG signal.

The equation below shows a basic EMG signal model, where $MUAPT_{j}$ is the motor unit action potential train, which refers to the collection of one motor unit positioned at the time of occurrence or separated by inter-discharge intervals, and *n*(*t*) denotes noise [7].

(1) $$\mathrm{EMG}\left(t\right)=\sum_{\;j=1\;}^{\;N_{m}}MUAPT_{j}\left(t\right)+n\left(t\right)$$

### 2.2. Noise and Denoising

During the detection and collection steps, there are many factors that can influence the collected signal. The denoising step is introduced in preprocessing to improve the quality of the signal. In this section, the main noise within a raw collected EMG signal is illustrated, and a denoising method is used.

#### 2.2.1. Noising

Initially, there is noise due to the instrument used to collect the EMG signal. First, for the two main kinds of EMG signal types, that is, the intramuscular EMG signal from the needle inserted into the muscle and the surface EMG signal from the surface electrodes, the sensitivity of the collection instruments, such as the needles and the electrodes, decides the noise that is collected with the EMG signal, which ranges from zero Hz to several thousand Hz; second, for the connection cable between the muscle, the electrodes and processing circuit, a slight movement will affect the electrical forces while the signal passes through [8]. By using better equipment and an intelligent circuit design, this noise can be efficiently reduced [8].

Second, there is noise generated by the human body. One type of noise is the electromagnetic noise that is generated by the body. The human body is similar to a small magnet that constantly generates electromagnetic radiation that propagates to the surroundings. This kind of noise is also known as ambient noise. The second type of noise comes from several factors, such as anatomical noise and biochemical noise, which take place in the biological unit of the detecting muscle; another type of this kind of noise is the “crosstalk” of unwanted EMG signals, which comes from other muscle tissues. Finally, the most interference comes from the heart’s electrical activity; another name for this is “electrocardiogram artefacts” [2,8]. These two types of noise can be reduced by a high-pass filter, because they are both generated due to random factors.

The last part is due to the instability of the signal, which is caused by the unstable firing rate of the motor unit, and, usually, these noise frequency components are approximately 0–19 Hz. The active motor unit and the muscle fiber mechanical reaction both play a large part in this noise [2].

In [7], the raw EMG signal was detected through an electrode and passed through a bandpass filter and a low-pass filter to reduce the temporal overlap, the number of superimposed waveforms and the amplitude of meaningless motor units. Additionally, nondiscriminative, low-frequency information is removed, which makes it easier to distinguish different motor units’ MUAPs, which are some of the features that can be extracted by reducing the variability of the shape of the signal.

#### 2.2.2. MPCA Denoising

The main denoising method that is used for EMG signals is the multiscale principal component method. Multiscale principal component analysis (MPCA) combines the features and capabilities of PCA and enhances the robustness of PCA in revealing hidden structures [9,10]. The main idea of PCA is to project N data points that have the mean value ***m*** and covariance matrix ***C*** onto the direction u to determine the direction that can maximize the project variance $u^{t}Cu$. To determine the maximum variance, we can set up an optimization problem that finds $\underset{u}{\max}u^{t}Cu$ under the condition $u^{t}u=1$ [9]. Hence, we can use the Lagrangian multiplier method, which is

(2) $$ℒ=u^{t}Cu-\lambda (u^{t}u-1)$$

Then, we differentiate ℒ, and when $\frac{dℒ}{du}=0$, we can obtain Cu=λu.

Another important part of multiscale PCA is the wavelet transform process. The general idea is that noise is a high-frequency signal that is normally distributed with a Gaussian distribution. As in the general model mentioned in the previous section, the EMG signal can also be described as

(3) F(t)=s(t)+e(t)

where ***s***(***t***) and ***e***(***t***) are the EMG signal and white noise, respectively, which means that the mean value of the noise is zero and the covariance is $\sigma^{2}$. Assuming the wavelet function is φ(t), $W_{e}\left(\alpha ,t\right)$ is the wavelet transform of *f*(*t*); hence, we have

(4) $$W_{e}\left(\alpha ,t\right)=\int_{-\infty }^{+\infty }e\left(u\right)\varphi_{e}\left(t-u\right)du$$

From this assumption, we can obtain the average power of $W_{e}\left(\alpha ,t\right)$,

(5) $$\begin{array}{ll} E\{{\left|W_{e}\left(\alpha ,t\right)\right|}^{2}\} & =\overset{+\infty }{\underset{-\infty }{\iint }}E\left\{e\left(u\right)e\left(v\right)\right\}\varphi_{a}\left(t-u\right)\varphi_{a}\left(t-v\right)dudv\\ & =\sigma^{2}\overset{+\infty }{\underset{-\infty }{\iint }}\delta \left(u-v\right)\varphi_{a}\left(t-u\right)\varphi_{a}\left(t-v\right)dudv\\ & =\sigma^{2}\int_{-\infty }^{+\infty }{\left|\varphi_{a}\left(t-u\right)\right|}^{2}du\\ & =\frac{\sigma^{2}{\left|\left|\varphi \right|\right|}^{2}}{a} \end{array}$$

We can determine that the average power of $W_{e}\left(\alpha ,t\right)$ is inversely proportional to the scale a [11].

The main process of MPCA is as follows [12,13]:

1. Use wavelet transformation to transform each channel of the EMG signal to the L of the decomposition level, and collect all the wavelet details in each channel into matrix $D_{L}$ and the wavelet approximations into matrix $A_{L}$.
2. Eigen-decompose the covariance matrix of each wavelet matrix, determine the eigenvector and eigenvalue of each, and then arrange them in descending order. The eigenvalues that select the threshold will determine the number of principal components that form a new matrix.
3. The relevance factor $\mu_{i}$ (*when two statistics, namely*, $T^{2}$*statistic and SPE statistic*, *exceed the control limits*, $\mu_{i}=1$ *otherwise*, 0) will select the nonsignificant scale and form a new EMG signal:
  (6) $$\;EM{G^{\prime }}_{i}=A_{L}+\sum_{i}^{L}\mu_{i}D_{L}$$

The two statistics and their control limit, which are used in Equation (6) to decide the value of $\mu_{i}$, are calculated as follows:

The $T^{2}$ statistic reflects the degree to which each principal component deviates from the model in terms of trend and amplitude; it is a measure of the internalization of the model, and it can be used to monitor multiple principal components simultaneously:

(7) $$T_{i}^{2}=X_{i}P_{k}\lambda^{-1}P_{k}X_{i}$$

(8) $$T_{\alpha }=\frac{k\left(m-1\right)\left(m+1\right)}{m\left(m-k\right)}F_{k,m-k,\alpha }$$

$T_{\alpha }$ is the control limit of the $T^{2}$ statistic, where *X* is the data matrix and *P* is the loading matrix; λ is the eigenvalue-diagnosed matrix, in which the eigenvalue belongs to the first *k* principal component; m represents the dimension of the data matrix *X*; and $F_{k,m-k,\alpha }$ is the *F* distribution at the significance level of α.

The *SPE* statistic describes the degree to which the measured value of the input variable deviates from the principal component model, and it is a measure of external variation in the model.

(9) $$SPE_{i}=X_{i}\left(I-P_{k}P_{k}^{T}\right)X_{i}^{T}$$

(10) $$SPE_{\alpha }=\theta_{1}{\left[\frac{C_{\alpha }\sqrt{2\theta_{2}h^{2}}}{\theta_{1}}+1+\frac{\theta_{2}h\left(h-1\right)}{\theta_{1}^{2}}\right]}^{\frac{1}{h}}$$

(11) $$\theta_{n}=\sum_{j=k+1}^{m}\lambda_{j}^{n}\;n=1,2,3\;$$

(12) $$h=1-\frac{2\theta_{1}\theta_{3}}{3\theta_{2}^{2}}$$

$SPE_{\alpha }$ is the control limit of the $SPE_{i}$ statistic, where $C_{\alpha }$ is the standard normal deviate corresponding to the upper (1−α) percentile.

### 2.3. Raw EMG Signal Processing

For a multichannel EMG signal, a necessary step is decomposition. By obscuring the composition of the EMG signal, we can easily determine that the EMG signal is composed of many MUAPs, so the features of classical techniques used for analysis, which are extracted from s-EMG signals, cannot guarantee effectiveness [14]. Therefore, if we can decompose the EMG signal, it will be easier to perform the next feature extraction step and make the features more representative.

#### 2.3.1. Independent Component Decomposition

In recent years, the independent component analysis (ICA) algorithm has appeared in many multi-dimension signal processing processes and mostly plays an important role, which is a special case of the blind source separation technique [8]. The main purpose of ICA is to transform an experimental multivariate random vector into independent sources [8]. The basis of the ICA is to normalize, whiten and iterate [5]. The whitening process uses a linear transformation to transform the components of the observed vector x into uncorrelated components with unit variance. After whitening, the parameters that need to be estimated in the ICA process will be reduced [5]. The iteration process is an algorithm used to calculate the independent components, and different algorithms have been used in this process, such as joint approximate diagonalization of eigenmatrices (JADE), the infomax estimation or maximum likelihood algorithm and the fast ICA algorithm [8]. We first discuss the standard ICA process. The standard process considers that the signal matrix x can be represented as an unknown, invertible, square matrix A with unknown pure signal vector s.

(13) x=As

We want to find an unmixing matrix w to obtain the following equation:

(14) s=wx=wAs

where wA=I [15]. The fast ICA algorithm is used to estimate matrix w. The fast ICA algorithm is based on a fixed-point iterative structure algorithm, and the goal is to make wx have the most non-Gaussian nature.

The first step of the fast ICA algorithm is to initialize the vector matrix w and then use g(u)=tanh(au) or other suitable functions as the derivative of the contraction function. Under the constraint condition $E\left\{{\left(wx\right)}^{2}\right\}={\left|\left|w\right|\right|}^{2}=1$, the optimal condition of E{G(wx)} can be obtained by the following formula:

(15) E{ xg(wx)}−βw=0

To use Newton iteration to solve this problem, we can obtain the vector matrix $w^{\prime }$, which is

(16) $$w^{\prime }=E\{xg\left(wx\right))\}-E\left\{g^{\prime }\left(wx\right)\right\}w$$

and then use

(17) $$w=\frac{w^{\prime }}{\left|\left|w^{\prime }\right|\right|}.$$

To calculate the new matrix, if the old *w* is in the same direction as the new *w*, then we can consider it convergent; otherwise, we calculate *w*′ iteratively. The obtained vector matrix is the matrix that we want, and based on this matrix, we can obtain the decomposed signal that we want [16].

#### 2.3.2. Wavelet Transform

The wavelet transform (WT) method is also a very common way to decompose a raw EMG signal. As a type of Fourier transform (FT), the WT is also a tool for time-frequency analysis; the difference between the WT and FT is that the WT possesses characteristics of multiresolution, which solves problems of the FT, such as the lack of localized analysis capabilities and the inability to analyze nonstationary signals [11].

The multiresolution theory means that when a scaling function $\left\{V_{j}\right\},\;\;j\in Z$ satisfies several conditions, it can be approximated by finite subspace $\left\{V_{j+1}\right\}$, which represents the low-frequency large-scale approximation part, and $\left\{W_{j+1}\right\}$, which represents the high-frequency detail part [11]:

(18) $$V_{0}=V_{1}⨁W_{1}=V_{2}⨁W_{2}⨁W_{1}=\cdots =V_{j}⨁W_{j}⨁W_{j-1}⨁\cdots W_{2}⨁W_{1}$$

The conditions that the scaling function need to meet are as follows:

1. $V_{j-1}\subset V_{j},\;j\in Z$;
2. $\cup_{j\in Z}V_{j}=L^{2},\;\cap_{j\in Z}\left\{0\right\}$;
3. $f\left(t\right)\in V_{j}\Leftrightarrow f\left(2t\right)\in V_{j+1}$;
4. $\forall k\in Z,\;\phi \left(2^{-\frac{j}{2}}t\right)\;\in \;V_{j}\Leftrightarrow \;\phi \left(2^{-\frac{j}{2}}t-k\right)\;\in \;V_{j}$;
5. $\exists \phi \left(t\right)\in V_{0}$, which make $\left\{\phi \left(2^{-\frac{j}{2}}t-k\right)-k|k\in Z\right\}$ become orthogonal.

The basic FT is expressed as

(19) $$F\left(t\right)=\int_{-\infty }^{+\infty }f\left(t\right)e^{-i\omega t}dt$$

The WT uses a basic wavelet φ(t) as the mother wave to replace the sinusoidal signal base, which is expressed as

(20) $$W\left(\alpha ,t\right)=\frac{1}{\sqrt{\alpha }}\;\int_{-\infty }^{+\infty }f\left(u\right)\varphi_{\alpha ,t}\left(\frac{u-t}{\alpha }\right)du$$

in which α and t are the scale and translation factors, respectively [11]. In practice, the discrete WT (DWT) is often used in the decomposition steps of the signal to discretize a continuous wavelet [1].

### 2.4. Feature Extraction Method

Feature extraction is a very useful way to transform a raw EMG signal into a set of features, and it is used by researchers to extract useful information in a signal and eliminate unnecessary information [17]. It is also very helpful for later classification. There are three main kinds of features: time-domain (TD) features, frequency-domain (FD) features and time–frequency-domain (TDF) features [17].

#### 2.4.1. Autoregression Coefficient

The autoregression (AR) coefficient is a time-domain feature that involves a linear combination of the previous signal x(n−1) to x(n−p). In this model, the EMG signal is described as a combination of the previous signal and noise:

(21) $$x\left(n\right)=\sum_{k=1}^{p}a_{k}x\left(n-k\right)+w_{n}$$

where $a_{k}$ is the autoregression coefficient and $w_{n}$ is white noise [18,19].

There are many mathematical methods to derive the autoregressive coefficients that define the autoregressive model, the most famous of which is the Yule–Walker model. This model uses the estimated values of the correlation function, which can be calculated as [20]

(22) $$r_{xx}\left(n,n-k\right)=E\left[x\left(n\right)x\left(n-k\right)\right]$$

Based on the above equation, the Yule–Walker matrix can be obtained:

(23) $$\left[\begin{array}{cccc} r_{xx}\left(0\right) & r_{xx}\left(-1\right) & \cdots & r_{xx}\left(-p+1\right)\\ r_{xx}\left(1\right) & r_{xx}\left(0\right) & \cdots & r_{xx}\left(-p+2\right)\\ \vdots & \vdots & \cdots & \vdots \\ r_{xx}\left(p-1\right) & r_{xx}\left(p-2\right) & \cdots & r_{xx}\left(0\right) \end{array}\right]\left[\begin{array}{c} 1\\ a_{1}\\ \vdots \\ a_{p} \end{array}\right]=\left[\begin{array}{c} \sigma_{m}^{2}\\ r_{xx}\left(1\right)\\ \vdots \\ r_{xx}\left(p\right) \end{array}\right]$$

where the value $\sigma_{w}^{2}$ is the variance of the model stochastic process. Hence, from the matrix, we can obtain

(24) $$r_{xx}\left(p\right)=r_{xx}\left(p-1\right)+a_{1}r_{xx}\left(p-2\right)+\cdots +a_{p}r_{xx}\left(0\right)$$

Since this matrix describes an equation system and fulfils the Toeplitz definition, the recursive Levinson–Durbin algorithm can be used to obtain the autoregressive coefficients $a_{p}$.

In the Toeplitz definition, the autoregressive coefficients $a_{p}$ can be obtained using the recursive Levinson–Durbin algorithm [20]. By observing the above matrix, we can see that this matrix fulfils the definition. Hence, we can use the mth-order Levinson–Durbin algorithm as follows to calculate the coefficients [21]:

(25) $$k_{m}=-\frac{\left[r_{x}\left(m\right)+\sum_{k=1}^{m}a_{k}r_{x}\left(m-k\right)\right]}{\rho_{m-1}}$$

(26) $$a_{m}\left(k\right)=a_{m-1}\left(k\right)+k_{m}\left(a_{m-1}\right)*\left(m-k\right),\;1\leq k\leq m-1,1\leq \;m\leq p$$

(27) $$\rho_{m}=\rho_{m-1}\left(1-k_{m}^{2}\right)$$

#### 2.4.2. The Decomposition Coefficient Features

In addition to the time-domain feature, the frequency-domain feature is an available component that can represent the information of the signal. Two types of features can be extracted based on the decomposition process: the first is the wavelet coefficient, and the other is the ICA coefficient. The wavelet coefficient is the feature vector of each decomposition scale, and the ICA coefficient is the weight of the decomposition signals.

### 2.5. Classification Method

#### 2.5.1. The K-Nearest Neighbor

There are many ways to classify EMG features, such as Bayesian techniques [22], the neural network method, the K-nearest neighbor (KNN) method [19] and the decision tree [23]. These methods all perform well in the classification process. Comparing several papers that use the matching learning tools implemented in calculating EMG features, KNN seems to be the simplest way to classify EMG features, and it can achieve relatively high accuracy. In reference [24], the author compared the artificial neural network (ANN) and KNN, and when the size of the feature set was the same, the operation time and accuracy of KNN were much better. Additionally, in reference [25], KNN and other classification methods were compared to classify the features in the time domain. Hence, in this paper, the KNN method is applied.

The KNN algorithm is a suboptimal process and is only useful when the dataset is relatively small. This algorithm assigns labels based on the k-nearest values of the data for classification. This distance is usually determined by Euclidean distance, and then iteratively updates by comparing new records with training records [23,26]. However, there are two issues to consider when using this algorithm. The first issue is that the algorithm assigns equal weights to each sample, which makes the labels difficult to determine when the sample set overlaps with the class labels. The second issue is that the strength of each classification sample cannot be determined [26].

#### 2.5.2. The Naïve Bayesian

The Bayesian aggregation has been used in many fields, such as medical and clinical applications. Generally, the probability of event A under the condition of event B is different from the probability of event B under the condition of event A; however, there is a definite relationship between the two, and Bayes’ rule is a statement of this relationship. The main function of the Bayesian aggregation is to use a priori numbers of occurrence and then combine it with specific sampled data values. As such, the Bayesian aggregation algorithm can produce an overall characterization [22].

## 3. Experiment

The experiment was conducted to determine how the denoising step influences the classification accuracy of the three features. The most important aspects of the experiment were to determine how denoising influences the signal in each processing step, what can be seen by illustrating every step and how each denoising process influences the feature extraction process.

### 3.1. The Dataset Used

**Figure 3:**
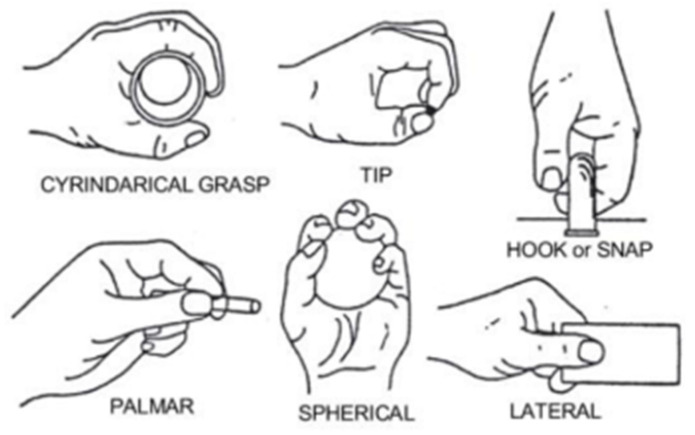
The grasping movements [27].

This research used an EMG signal database downloaded from the UCL machine learning website. This dataset was collected using LabVIEW with national directives as the core, and it has been widely applied in many research papers that study EMG signals. This dataset has two different data groups with same six grasping movements see as Figure 3, and the EMG signals of each data group were collected differently. The sampling rate of the EMG signal was 500 Hz, and a Butterworth bandpass filter with a range of 15–500 Hz and a notch filter with a bandstop of 50 Hz were applied to eliminate the artefacts of signals caused by interference, such as those from wires.

The EMG signal was taken from two forearm surface EMG sensors, meaning that the signal is a two-channel EMG system. There are two kinds of datasets in the UCL machine learning repository: the one used in this experiment was collected from five people, three of whom are female and the other two of whom are male. This dataset was formed by collecting the data of the people performing six full grasping movements thirty times each, and the signal collected from each person had its own mat file. These six grasping movements are shown below.

### 3.2. Comparison of The Denoising Signal and Original Signal

The figure shows the MPCA process that was performed on the two different channels, which calculates the average of all 30 signals in one channel. The MPCA algorithm is based on MATLAB and can be accessed worldwide. The basic wavelet used in this process is the Daubechies least asymmetric wavelet, and a heuristic rule is used to determine how many principal components are retained. The heuristic rule ensures that the component associated with the eigenvalues is greater than 0.05 times the sum of all eigenvalues.

**Figure 4:**
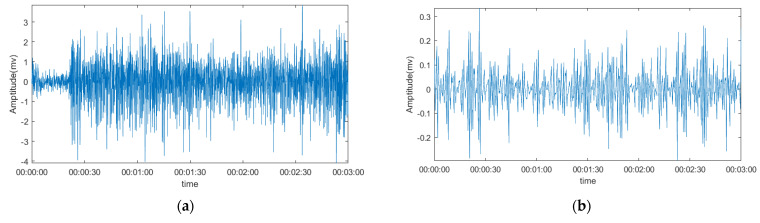

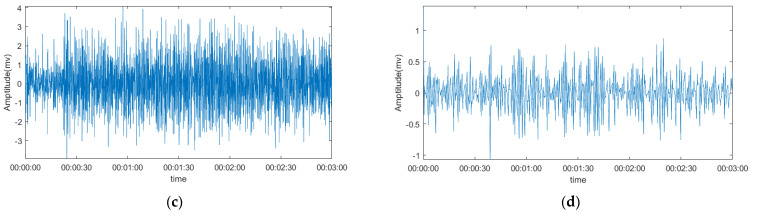
The outcome of the MPCA denoising process. (**a**) The original signal of channel 1. (**b**) The denoising signal of channel 1. (**c**) The original signal of channel 2. (**d**) The denoising signal of channel 2.

Because the signal gathers in two different channels, we want to see if there are any differences in denoising for these two channels’ signals. From Figure 4, we can see that, despite the slight differences, these two channels’ signals do not show many differences; they all seem to process well. By comparing the right column and left column, which means the signal with or without denoising, we can see that there are two improvements in the MPCA denoising method. The first improvement is that the density of the signal is decreased, which is because when using denoising steps, less relative information is removed. The second improvement is that the MUAPTs, which formed as a result of the hand movement, are more easily to distinguish by the amplitude change.

**Figure 5:**
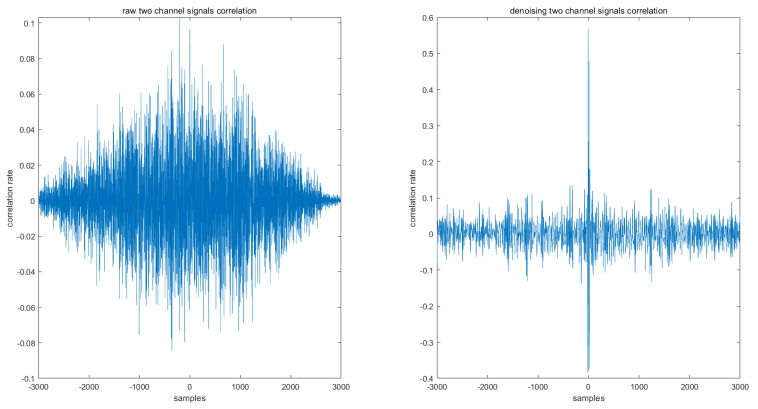
The correlation between the two channels.

The correlation between the two channels can be calculated with the following equation:

(28) $$R_{xy}\left(m\right)=E\left\{x_{n+m}y_{n}^{*}\right\}=E\left\{x_{n}y_{n-m}^{*}\right\}$$

where *x* and *y* are the signals, and the correlation vector of the raw signal and the denoising signal is shown in Figure 5. As an observation, the correlation between the two channels improved.

### 3.3. Signal Decomposition

#### 3.3.1. Wavelet Decomposition

In this experiment, a four-level wavelet decomposition of the EMG signal using the fourth-order Daubechies wavelet was implemented. The coarse-scale approximation coefficients and the detailed coefficients from the decomposition are shown in the upper figure.

**Figure 6:**
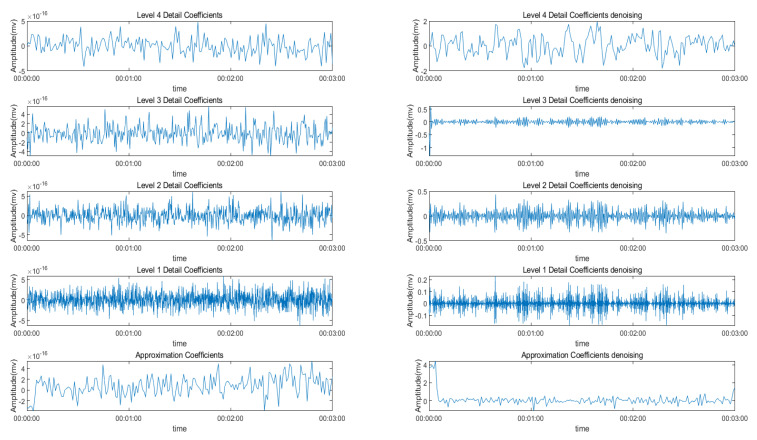
The wavelet decomposition coefficient feature vectors at different levels.

Figure 6 shows the detail coefficients and the approximate coefficient of the denoising signal and the raw EMG signal. The difference between these two kinds of signal is obvious. The denoising signal shows a more clear change in aptitude, which is associated with the hand movement and is similar to the denoising signal in the first denoising step.

**Figure 7:**
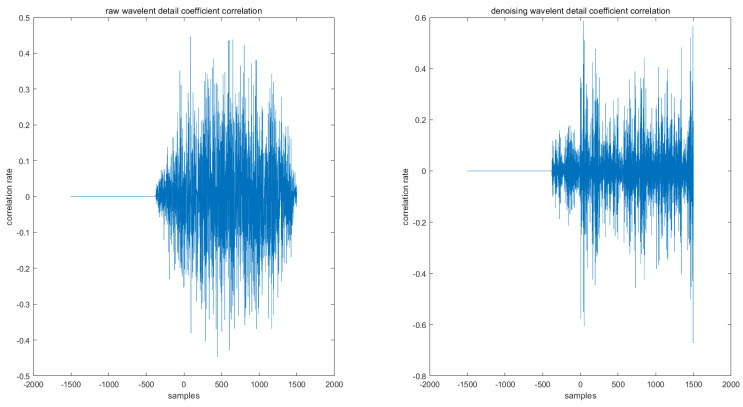
The correlation between the different levels of wavelet detail coefficients.

As can be seen in Figure 7, by calculating the correlation rate of the different levels of detail coefficients, the correlation between the different levels of wavelet detail coefficients increases after denoising.

#### 3.3.2. ICA Decomposition

**Figure 8:**
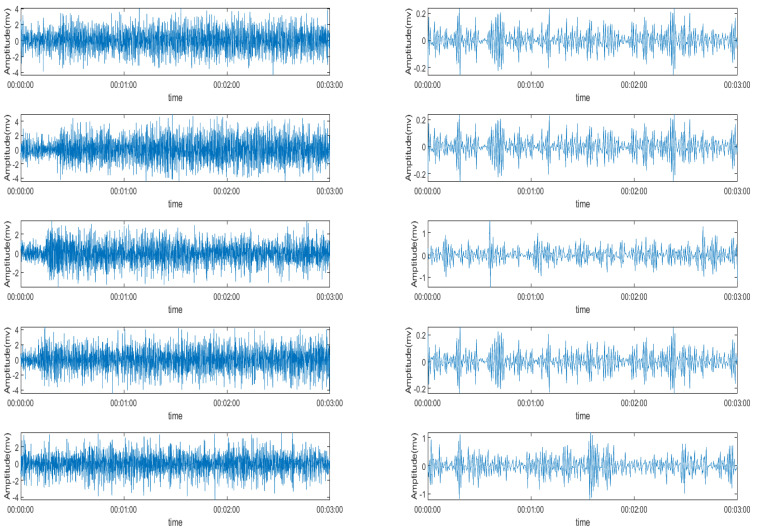
The ICA decomposition signals.

Here, in the ICA decomposition steps which shows an example in the Figure 8, the construction function is used as the smoothing function, which is similar to the absolute value function and has four decomposition signals.

As well as the decomposition incorporation of the two kinds of EMG signals, these signals, when going through the ICA decomposition steps, also show that the denoised EMG signal has a clearer boundary between each MUAPT. However, different from the wavelet decomposition, the feature vector that the ICA method decomposed, the raw EMG signal seems to correlate more to each other and the original signals.

In these two different types of decomposition functions, we can see that the signal decomposed into several separate signals, which can extract features that are more representative than the original signal, and the denoising step clarified the features of the recomposited signal.

### 3.4. Features

There are three kinds of features that are extracted from the EMG signal: the first is the autoregression coefficient, which is calculated by the Yule–Walker method, and the other two are the coefficients generated by the decomposition steps as illustrated above. Here, the wavelet decomposition decomposes the EMG signal at level 9, and the detailed coefficient extracted by each level scale occupies a frequency band. The energy of each scale, which is calculated by the quadratic sum of all the coefficients in the scale, is selected and forms the feature vector DWTF.

$$ar_{coeff}=\left[ar_{1},ar_{2},ar_{3},ar_{4},ar_{5},ar_{6},ar_{7},ar_{8},ar_{9},ar_{10}\right]$$

$$ICAF=\left[ica_{1},ica_{2},ica_{3},ica_{4},ica_{5},ica_{6},ica_{7},ica_{8},ica_{9},ica_{10}\right]$$

$$DWTF=\left[ca_{9},cd_{9},cd_{8},cd_{7},cd_{6},cd_{5},cd_{4},cd_{3},cd_{2},cd_{1}\right]$$

### 3.5. Classification

In the classification step, the features are separated into ten folds, and the cross-validation method is used to calculate the correction rate of the classification model. The correction rate is calculated by the following formula:

(29) $$R=\frac{N_{tp}+N_{tf}}{N_{t}}\times 100\%$$

where $N_{tp}$ is the number of features that are classified as the correct class, $N_{tf}$ is the number of features that are classified as the wrong class, and $N_{t}$ is the total number of features that participate in this classification process.

The content and meaning of Table 1, Table 2, Table 3, Table 4 and Table 5 are shown next. Table 1 and Table 4 show the two different classification results of the three different classification methods. In Table 1, by using the K-nearest neighbor, the denoising only shows some improvement in the ICA coefficient and makes the classification correct rate of the autoregression coefficient and the WT coefficient, the confusion matrix of each classification result are illustrated in Table 2, Table 3 and Table 5. However, in Table 4, the naïve Bayesian classification method shows a different result of the wavelet transform coefficient; the denoising step increased a lot of the classification accuracy of this feature.

The difference between the KNN and the naïve Bayesian is that the classification method is different. The basic concept of the KNN is based on the distance between each sample, which is based on the Euclidean distance, but the naïve Bayesian is based on the probability of each category appearing under the condition that this item appears, whichever is the largest, considering which category the item to be classified belongs to. The differences in the classification methods also influenced the denoising method. However, in both classification methods, the MPCA has a negative influence on the classification accuracy rate of the time-domain feature—the autoregression coefficient.

## 4. Discussion

In this experiment, several features were extracted from a raw EMG signal. The autoregression coefficient is a time-domain feature that represents the time connection of one signal. Independent component analysis and wavelet transform analysis describe a signal in the frequency domain; they decompose the signal into different scales, and ICA decomposition separates the signal into different signals that show the components of the original signal. Wavelet transform uses different basic wavelet functions to separate the signal to simulate the original one. There are some similarities between the two different decomposition methods, such as the fact that they are both frequency-domain feature extraction methods and they both divide the original signal into different levels of decomposition signals. By judging the classification accuracy and confusion matrix of the two, we can see that the features formed by the wavelet decomposition method show better results in both classification methods. The effect of wavelet denoising for ICA decomposition is much better than that of wavelet decomposition. In both classification methods, the features of the ICA decomposition method significantly improved after wavelet denoising.

In the KNN classification algorithm method, different neighbor numbers affect the correction rate, so different neighbor numbers were tested in this paper from K = 1 to K = 10 by 3 to test which value of k is suitable for classification. Moreover, we can see that when the value is 7, the classification accuracy of the ICA coefficient feature and the autocorrelation feature reach the maximum point. However, the WT coefficient feature reaches the maximum point when k = 1.

The results are shown in the Classification Section. By judging the information in Table 1, Table 2, Table 3, Table 4 and Table 5 in Section 3.4, we can see that the classification accuracy rate of the autoregression coefficient feature shows a huge decrease, which only successfully classifies half of the features compared to the one that uses the original signal to carry out the feature extraction. The Bayesian method showed a poor accuracy rate, and none of the features expected in the naïve Bayesian classification method exceeded 50%. The denoising step improved the classification rate of the ICA features but showed a worse result in the classification of the other two features. The reason, we believe, is that multiscale PCA uses wavelet transform for denoising, so the denoising process is more efficient in the frequency domain, and this process can erase some of the important relationships in the time series, so the autoregression coefficient shows worse behavior after the denoising process. For the wavelet decomposition coefficient, we believe the reason why the classification behaves worse is that the wavelet transform was already used in the denoising step.

This study has several limitations, including using too-few EMG signal data. Although our findings indicate that MPCA can improve the classification rate of ICA features with the KNN method and naïve Bayesian classification method, more empirical studies on the denoising steps need to be conducted in future tests, such as using the naïve Bayesian classification method.

In the research process, we reviewed many papers, and much of the research relates to electroencephalograms (EEGs). EEG signal processing is very popular and is similar to EMG signal processing. Additionally, the brain–computer interface, a concept that has attracted our attention, is highly relevant to these two signals. EEG signals have low accuracy, and some patients suffer from neuromuscular disease, which means that the EMG signals are limited [28]. By combining these two signals, we can acquire more accurate muscle movement for applications such as typing without hands. In future studies, we would like to study the difference between these two signals and find a way to make use of both of them.

## Figures and Tables

**Table 1:** The KNN classification results.

*Features*	Multiscale PCA Denoising				Original Signal			
	k = 1	k = 4	k = 7	k = 10	k = 1	k = 4	k = 7	k = 10
*WT coefficient*	100%	80.27%	46.77%	53.33%	100%	85.05%	65%	74.5%
*ICA coefficient*	53.66%	47.88%	48.16%	46.61%	42.27%	38.16%	36.33%	33.05%
*Autoregression coefficient*	33.72%	33.27%	34.44%	31.88%	69.11%	72.38%	73.88%	73%

**Table 2:** The confusion matrix of the KNN method—denoising part.

*Features*	Multiscale PCA Denoising			
	k = 1	k = 4	k = 7	k = 10
*WT coefficient*	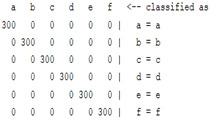	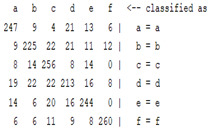	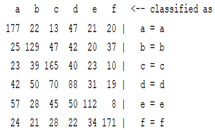	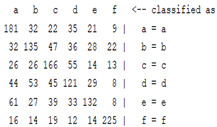
*ICA coefficient*	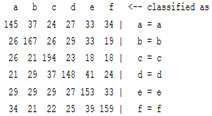	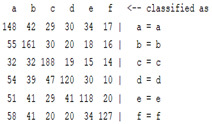	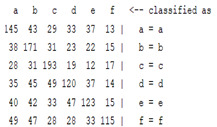	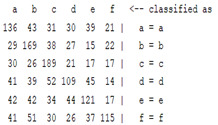
*Autoregression coefficient*	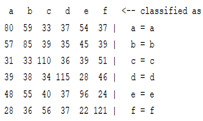	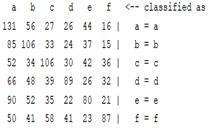	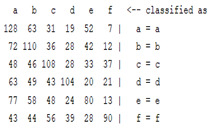	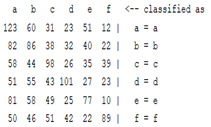

**Table 3:** The confusion matrix of the KNN method—raw signal part.

*Features*	Original Signal			
	k = 1	k = 4	k = 7	k = 10
*WT coefficient*	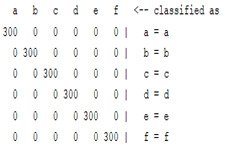	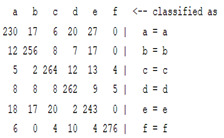	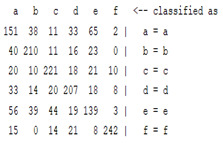	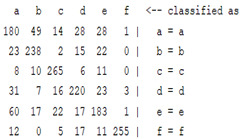
*ICA coefficient*	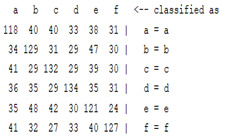	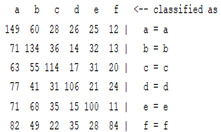	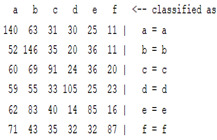	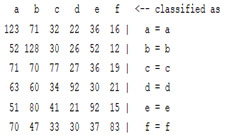
*Autoregression coefficient*	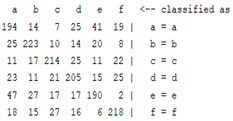	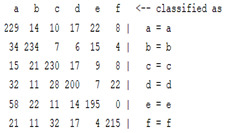	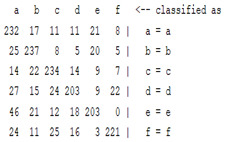	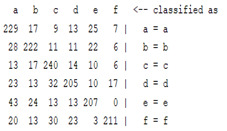

**Table 4:** Classification with the naïve Bayesian method.

*Features*	Multiscale PCA Denoising	Original Signal
*WT coefficient*	22.72%	46%
*ICA coefficient*	15.27%	8.27%
*Autoregression coefficient*	22.38%	43.5%

**Table 5:** The confusion matrix of naïve Bayesian method.

*Features*	Multiscale PCA Denoising	Original Signal
*WT coefficient*	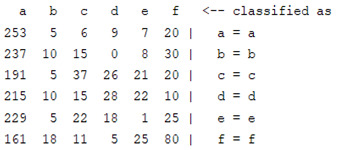	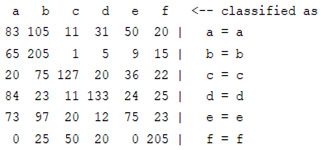
*ICA coefficient*	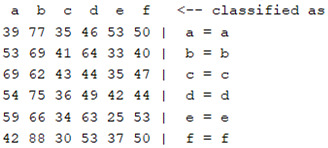	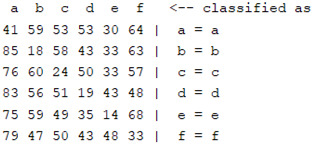
*Autoregression coefficient*	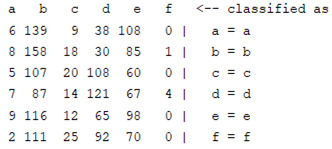	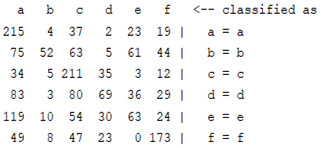

## Data Availability

The data presented in this study are openly available in [UCI Machine Learning Repository] at [https://archive.ics.uci.edu/ml/datasets/sEMG+for+Basic+Hand+movements], reference number [27].
